# Mass vaccination, immunity and coverage: modelling population protection against foot-and-mouth disease in Turkish cattle

**DOI:** 10.1038/srep22121

**Published:** 2016-02-26

**Authors:** T. J. D. Knight-Jones, S. Gubbins, A. N. Bulut, K. D. C. Stärk, D. U. Pfeiffer, K. J. Sumption, D. J. Paton

**Affiliations:** 1 The Pirbright Institute, Pirbright, United Kingdom; 2 The Royal Veterinary College (VEEPH), University of London, United Kingdom; 3 The Şap Institute, Ankara, Turkey; 4 The European Commission for the Control of FMD, FAO, Rome, Italy

## Abstract

Foot-and-mouth disease (FMD) in Turkey is controlled using biannual mass vaccination of cattle. However, vaccine protection is undermined by population turnover and declining immunity. A dynamic model of the Turkish cattle population was created. Assuming biannual mass vaccination with a single-dose primary course, vaccine history was calculated for the simulated population (number of doses and time since last vaccination). This was used to estimate population immunity. Six months after the last round of vaccination almost half the cattle aged <24 months remain unvaccinated. Only 50% of all cattle would have received >1 vaccine dose in their life with the last dose given ≤6 months ago. Five months after the last round of vaccination two-thirds of cattle would have low antibody titres (<70% protection threshold). Giving a two-dose primary vaccination course reduces the proportion of 6–12 month old cattle with low titres by 20–30%. Biannual mass vaccination of cattle leaves significant immunity gaps and over-reliance on vaccine protection should be avoided. Using more effective vaccines and vaccination strategies will increase population immunity, however, the extent to which FMD can be controlled by vaccination alone without effective biosecurity remains uncertain.

Vaccines play a crucial role in the control of foot-and-mouth disease (FMD) and are widely used throughout the world^1^. Whilst FMD has been eradicated in Turkish Thrace, mass vaccination has failed to control the disease in the rest of the country (Anatolia, Fig. 1). FMD vaccination in Turkey typically consists of biannual mass vaccination of cattle. Most farms in Anatolia are smallholdings dependent upon communal grazing, and vaccination should be performed before animals are turned out for spring grazing and at the end of the grazing season in autumn, with cattle typically housed over winter.

Within Turkey, there is great variation in topography, climate and livestock husbandry. This results in differences in the seasonality of livestock births and population age structure. These demographic factors determine the proportion of cattle eligible for routine FMD vaccination (≥2 months old), the proportion recently vaccinated and the proportion that have received multiple doses. These proportions change with time as animals are born, age and die. However, population structure is rarely considered in detail during livestock vaccination programmes^2^.

The trivalent vaccine used in Turkey at the time of the study was reported to be ≥3PD_50_, covering serotypes O, A and Asia-1. The 50% protective dose (PD_5o_) is a measure of vaccine potency assessed in a challenge study. A single dose of a ≥3PD_50_ vaccine contains at least three times the dose required to prevent clinical FMD in 50% of cattle that have FMD virus injected into the tongue three weeks after vaccination.

Quality FMD vaccines can induce immunity lasting for ≥6 months after a single dose. Immunity is broader and longer-lasting with a faster onset if vaccine potency is high^3^,^4^,^5^,^6^. However, many FMD control programmes use vaccines with a shorter duration of immunity and several doses of vaccine are required before protection is sustained^7^,^8^,^9^. When first vaccinated, cattle should receive two doses of vaccine approximately one month apart (a two-dose primary course)^5^,^7^,^10^. However, to save resources, a single-dose primary course is used in many countries, including Turkey at the time of this study.

We previously assessed immune response in Turkish cattle after routine FMD vaccination under field conditions^8^. However, that study only assessed a small subgroup of the vaccinated population and did not take into account age structure of the population at large and population turnover. In addition, the vaccine history of those sampled did not reflect that of the population at large.

The objective of this modelling study was to quantify population immunity resulting from mass vaccination of cattle in Turkish Anatolia, using data from 2012/13. As we wished to evaluate protection provided by the vaccination programme, immunity from natural infection was not considered. Percentage vaccinated and vaccine immunity were modelled over the annual production cycle. The effect and cost-effectiveness of a single versus a two-dose primary course were estimated.

## Materials and Methods

Referenced field studies obtained ethics approval from the University of London and The Pirbright Institute. Methods were carried out in accordance with approved guidelines.

We developed a simulation model of FMD population immunity in Turkish cattle. This was undertaken by combining a dynamic model of population structure and vaccine coverage with estimates of post-vaccination immune response^8^. Two main sources of data were used to inform the model: (a) demographic data from nationwide cattle surveys, and (b) an extensive field study of post-vaccination serology^8^.

**Figure 1:**
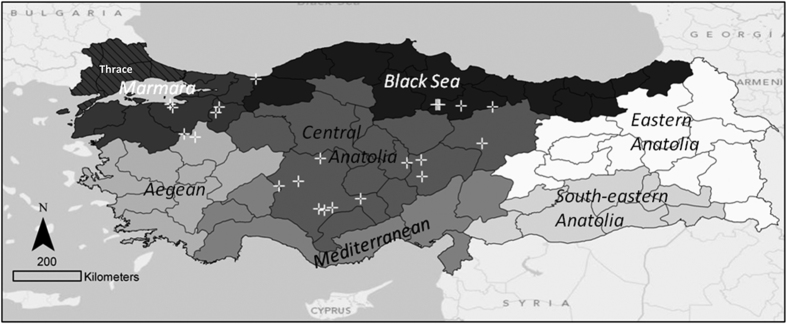
Map of Turkey. The locations of the 23 villages included in the prospective serological field survey used to inform the model in this study are marked with crosses^8^. The hashed lines show the FMD-free with vaccination zone of Thrace. Turkey consists of seven regions, divided into 81 provinces and 957 districts, containing about 48,000 villages. Created using ArcGIS® software by Esri (ArcMAP10.3).

Maternally derived immunity and immunity from infection have not been considered. The study assessed Anatolia and not FMD-free Turkish Thrace where different vaccines are used (Fig. 1).

### Cattle population model

The population model was designed to represent cattle age structure in each province. The number of cattle <12 months, 12 to <24 months and ≥24 months on 31^st^ December 2012 was obtained for each sex in each province from government census records^11^. Demographic data taken from separate randomised cross-sectional surveys of cattle, conducted in 2009, 2010 and 2012, covering all provinces, were used to estimate month of birth within age categories. The FMD sero-surveys sampled 96 249 cattle aged 6–24 months^12^. For each of the 78 provinces in Anatolia, survey data from cattle 6–17 months old were used to create a distribution of month of birth (Supplementary Dataset, month_of_birth_2.xls). Each distribution was then sampled *n* times, where *n* was 1% of the number of cattle within each age category for each province. This gave a simulated population of 135 453 cattle representing a random selection from across Anatolian Turkey.

Having sampled the month of birth for the simulated population, the actual day of birth was selected at random from a uniform distribution (e.g. from 1–31 for January). As the age distribution of cattle ≥24 months old was not available, those cattle ≥24 months old were assumed to be equally distributed between the ages of two and five years old.

The simulated population reflected the age-sex structure for each province on 31^st^ December 2012.

### Vaccine coverage

Once the cattle population was simulated, the vaccination history of each animal was derived assuming nationwide biannual mass vaccination since 2007 on the 25^th^ March and 25^th^ September each year (the 2012 average date of autumn vaccination). If a simulated animal was ≥2 months old on a vaccination date it was eligible for vaccination.

To incorporate realistic levels of vaccine coverage (the proportion of eligible cattle vaccinated), district coverage was assumed to vary (Anatolia, Turkey has 904 districts in 78 provinces). For each district in the simulated population, the proportion of eligible cattle typically vaccinated at mass vaccination (*vc*) was sampled from a betapert distribution (minimum = 40%, maximum = 100%, most likely = 80%), based on Turkish field studies^13^. Whether or not an eligible animal was actually vaccinated, during a particular round of vaccination, was then determined by a Bernoulli distribution, with probability of vaccination *vc*.

### Population immunity

#### SP prediction models

Antibody levels against FMD virus structural proteins (SP) are a strong correlate of protection and can be measured to assess FMD immunity^14^,^15^,^16^,^17^,^18^. Log_10_ SP antibody titres for serotypes A, O and Asia-1 were predicted for each animal in the simulated population. SP titre measured by liquid phase blocking ELISA (LPBE) was predicted in two steps using regression models fitted to data from an extensive post-vaccination field study performed in Turkey^8^. Predictor variables were 1) number of vaccine doses received in a lifetime (which was correlated with age), 2) time since last vaccination and 3) was a single or two-dose primary course given^8^.

Firstly, a GEE (generalised estimator equation) logistic regression model predicted the probability of an animal having a titre equal to or higher than 1:32, which was the lowest dilution tested. This probability was then used as the parameter in a Bernoulli distribution.

In the second step, for each animal predicted to have a titre above the detection threshold (≥1:32), an interval regression model was used to determine expected Log_10_ (SP titre). For these cattle, final predicted SP titre was determined by drawing a sample from a normal distribution centred on this expected titre, with standard deviation equal to the model residual standard deviation^19^.

Available data only described the effect of the two-dose primary course on immunity following initial vaccination. Data showing its effect on immunity after further six-monthly rounds of vaccination were not available^8^. Therefore, the regression coefficient for the two-dose primary course was only applied to simulated cattle at first vaccination. After further rounds of vaccination the two-dose primary course was modelled as the effect of having received an extra dose of vaccine in an animal’s lifetime.

Almost all adult cattle >24 months would have been vaccinated ≥3 times. As cattle vaccinated ≥3 times are usually able to sustain antibody titres throughout the duration of the six month inter-vaccination interval they were combined in one group^8^. Unvaccinated cattle were assigned a titre of zero.

#### Modelling uncertainty

To account for variability and uncertainty, Monte Carlo simulation was used. The population model was simulated 1000 times. For each iteration, the regression parameters used for prediction were sampled from normal distributions with mean equal to the regression coefficient and standard deviation equal to the robust standard error. SP titres were then predicted for each iteration and summary statistics were produced, including the proportion of the simulated population with a titre <1:10^2^. In challenge studies animals with a titre ≥1:10^2^ are likely to be protected against homologous challenge (personal communication A.N. Bulut), with approximately 70% protected^20^,^21^,^22^.

To assess the accuracy of SP predictions, titres were predicted for the cattle in the field study used to fit the regression models^8^. These predicted titres were then compared with actual titres for the same animals. Analysis was performed using R^23^. In the results, median predicted values are shown with 95% prediction intervals (PI), i.e. 2.5^th^ to 97.5^th^ percentiles.

### Coverage and immunity over time

The population model was used to assess vaccination and immune status on 25^th^ October 2012 (one month post-vaccination, when antibodies peak) and 14^th^ February 2013 (the maximum time after vaccination that antibody titre was assessed in the post-vaccination field study used to fit the prediction models^8^). Vaccination and immune status were also assessed on the date of autumn and spring vaccination, (25^th^ September 2012 and 25^th^ March 2013). However, as the time since last vaccination on these dates is greater than in the data used to fit the regression models, caution is required when assessing antibody levels on these dates.

Assessment of the relationship between percentage vaccinated and population immunity is presented in the Supplementary Methods and Results.

### Vaccine homologous antibody

Antibodies in post-vaccination sera bind vaccine homologous virus better than they bind other strains of FMD virus. The vaccine in the field study used to fit prediction models was the trivalent FMD vaccine produced by the Sap Institute in Ankara, Turkey, containing FMD strains O Panasia II [O Tur 07], A Iran 05 [A TUR 06] and Asia-1 Sindh-08 [Asia-1 TUR 11]. The antigens used in the LPBE (O Manisa, A22 IRQ 24/64 and Asia-1 Shamir) are all different to the antigens used in the vaccine^8^. Therefore, virus neutralisation (VN) tests were used to estimate the effect of this antigen mismatch on predicted protection as described by Knight-Jones *et al.* (2015).[Table t1]

The proportion protected was then adjusted according to the difference in the proportion above the 70% protection threshold according to VN and LPBE tests. This was done separately for simulated LPBE titres below the detection threshold, detectable but <1:10^2^ and finally those ≥1:10^2^. Adjustments were made using Betapert distributions based on the median, 2.5^th^ and 97.5^th^ percentiles shown in Table 2 of Knight-Jones *et al.* (2015), which represent the proportion above the homologous VN 70% protection threshold according to LPBE titre.

### Cost-effectiveness of the two-dose primary course

The additional cost of routinely using the two-dose primary course was estimated by multiplying the number of cattle vaccinated for the first time at spring and autumn by the estimated cost of vaccination (vaccine plus administration) taken as Betapert (min = US$0.4, max = US$3, most likely = US$1), based on costs in Turkey and elsewhere^1^,^24^,^25^,^26^. The cost-effectiveness of the strategy was estimated as the increase in the percentage of cattle with a titre above the 70% protection threshold for each additional US$100,000 in vaccination costs. This was assessed after adjustment to reflect protection against homologous challenge assessed by VN for serotype O only.

Calculations for the two-dose primary course included administering the two-dose primary course to all cattle when first vaccinated, including those that were not presented for vaccination as calves.

## Results

### Population structure

**Figure 2:**
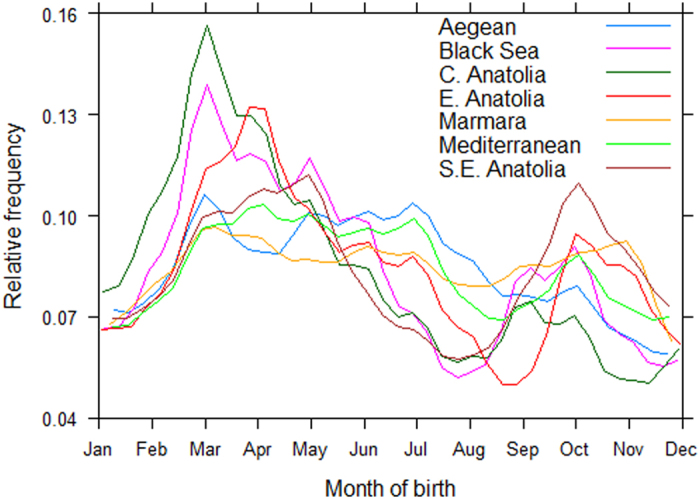
Relative frequency of births over the year for cattle from different regions of Turkey. Taken from month of birth of cattle included in three Anatolian FMD random sero-prevalence surveys (2009, 2010 and 2012), n = 96 249. Coastal regions, except for the mountainous Black Sea region, experience less seasonal temperature variation and had more uniform birth rates throughout the year. Spring/summer calving is common in Central and Eastern Anatolia, where winter temperatures often average below 0 °C.

The proportion of births occurring in a particular month varied by region (Fig. 2). Age distribution details are shown in Table 1 and Supplementary Table S1, and Fig. S1.

### Vaccine coverage

When incomplete coverage of eligible cattle was incorporated, about 20% of cattle were unvaccinated six months after the last round of vaccination. An additional 15% of cattle, although vaccinated, had not been vaccinated for a year or more. Only about half of all cattle would have been vaccinated ≥3 times since birth (Table 1). Looking at cattle <12 months old, about 82% had not previously been vaccinated at the time of autumn vaccination, with about 69% unvaccinated at the time of spring vaccination, the difference resulting from more calves being born in spring/summer than in autumn/winter. For all cattle aged <24 months, 44% would be unvaccinated at the time of autumn vaccination.

Even if all of the 94.7% of cattle ≥2 months old were vaccinated in spring 2012, then six months later, new births would have increased the percentage of unvaccinated cattle to 18.5% (Table 1 and Fig. S2).

### Post-vaccination SP titre

**Figure 3:**
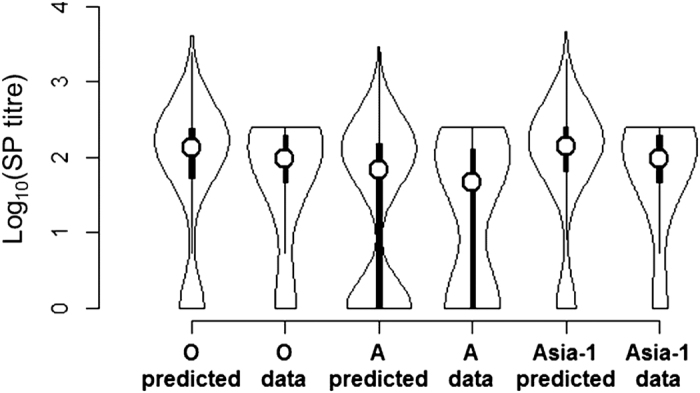
Violin plot showing actual SP titres after FMD vaccination (labelled “data”) and predicted SP titres for the same cattle (serotypes O, A, Asia-1). Predictions are on a continuous scale to remove interval and right censoring in the original titres. Data collected in a prospective field study in Turkey^8^ (see Fig. 3 of Knight-Jones *et al.* (2015) for details). The proportion of cattle with a particular titre is proportional to violin-plot width. Box-plots within the violin-plot show median (white circle), inter-quartile range, minimum and maximum titres. Actual titres (“data”) below the detection threshold (1:32) were designated zero.

The coefficients for the regression models used to predict SP titre are shown in Table 2. Titre increases with prior vaccination and a two-dose primary course, declining with time since vaccination. Figure 3 shows that the two-step modelling process accurately recreated the original antibody distribution. As right and interval censoring, present in the original serial dilution data, were removed in the predicted titres, the latter were slightly raised without truncation at the maximum test dilution.

Population antibody levels were highly bi-modal (Fig. 3 and Supplementary Fig. S3). Many cattle were below the antibody detection threshold of 1:32, the rest had SP titres that varied around an average of roughly 1:10^2^.

Only at peak antibody response, one month after the last round of vaccination, did more than half the simulated cattle population have a titre ≥1:10^2^ (Table 3). A quarter had no detectable antibodies at this time of peak response. Antibody levels were lower for serotype A and largely similar for O and Asia-1. Low serotype A titres were partly a result of differences in vaccine and LPBE antigen (see VN adjustments later).

By February, one month before re-vaccination, 68% (95% PI: 60–75%) of cattle had a serotype O SP titre of below 1:10^2^. Using a two-dose primary course resulted in only an additional 8% having a titre above this threshold. However, the beneficial effect occurred mainly in young animals. By mid-February, with a single-dose primary course, roughly 80% of 6-<12 month old cattle had titre <1: 10^2^ compared to 50–60% with the two-dose primary course (all serotypes). An additional 20–30% were also lifted above the detection threshold (1:32).

### Vaccine homologous antibody

Compared to LPBE estimates, protection against vaccine homologous virus was higher when predicted by VN except for Asia-1. After adjusting LPBE protection estimates to reflect protection against vaccine homologous virus predicted by VN, assessed on 25^th^ October 2012 (one month post-vaccination), 26% [95% PI: 19–34%], 37% [95% PI: 28–48%] and 59% [95% PI: 46–72%] of cattle were expected to be below the 70% protection threshold for serotypes O, A and Asia-1 respectively. The same statistic assessed in mid-February would yield 44% [95% PI: 33–56%], 48% [95% PI: 38–60%] and 74% [95% PI: 60–83%] below the threshold with a single-dose primary course and 39% [95% PI: 29–51%], 44% [95% PI: 34–56%] and 71% [95% PI: 58–81%] if a two-dose primary course were used, again for serotypes O, A and Asia-1, respectively.

### Cost-effectiveness of the two dose primary course

Looking at serotype O only, after autumn vaccination, by mid-February, the two-dose primary course increased the proportion above the 70% protection threshold, adjusted to represent homologous challenge using VN, from 56% [95% PI: 44–67%] to 61% [95% PI: 49–71%]. The total cost of administering an additional vaccine dose to the 1.9 million cattle first vaccinated in autumn 2012 would be US$2.3million [95% PI: US$ 2.3 to 2.4million]. This equates to an extra 0.2% [95% PI: 0–0.8%] above the 70% protection threshold per US$100,000 of additional vaccination costs.

Routine use of the two-dose primary course increased the proportion of cattle aged 6-<18 months old with predicted titres above the 70% protection threshold from 51% [95% PI: 39–63%] to 66% [95% PI: 58–74%]. This equated to an additional 0.7% [95% PI: 0.1–1.3%] of this age group above the threshold for every additional US$100,000 spent.

## Discussion

### Key findings

In this study, we extended the serological approach to FMD post-vaccine monitoring, by incorporating estimates of immune response into a dynamic, demographic model of vaccine coverage. The model predicted that the FMD vaccination programme in Anatolia provided only limited protection against FMD. Although the vaccination programme uses biannual mass vaccination of cattle, five months after the last round of vaccination, half to two-thirds of cattle would have low antibody titres.

#### Declining antibodies and the need for multiple doses

Time since last vaccination and the number of animals that had received three or more vaccinations in their life had a large effect on predicted immunity. With six-monthly vaccination and a single-dose primary course, sustained protection could not be achieved at a young age due to rapidly declining antibodies and the need for multiple doses. Vaccines that induce sustained immunity after one or two doses are required. The vaccines assessed in this study are reported to be ≥3PD_50._ Although few data are available, following a single dose with a ≥6PD_50_ vaccine, antibody levels have been observed to remain high for six months or more^7^,^27^. Under this scenario protection levels would mirror the proportion vaccinated with minimal decline in immunity for the next six months or more. Higher potency vaccines are also more likely to protect in the event of poor vaccine match^6^.

#### Two-dose primary course

Considering cattle <12 months old, five months after the last round of vaccination about 80% would have low titres. Using a two-dose primary vaccination course improved immunity in young animals to levels similar to adults; it also provides an additional opportunity to vaccinate young-stock previously missed. Young cattle experience a high incidence of FMD^13^. Although the two-dose primary course requires significant additional resources (about 20% more doses), reducing susceptibility of young-stock as soon as possible is vital.

#### Clusters of low coverage

Six months after the last round of vaccination about 20% of cattle would be unvaccinated with only around 50% vaccinated more than once in their life, with the last dose received ≤ 6 months ago. Two-thirds of cattle <12 months old may remain unvaccinated when turned out to communal grazing at spring. This results from both a failure to vaccinate cattle that should be vaccinated and the presence of animals that at the previous round of vaccination were either too young for vaccination (<2 months) or not yet born.

Temporo-spatial variation in these factors will lead to clusters of high susceptibility. Mass vaccination, with a one-size-fits-all approach, is particularly vulnerable to this phenomenon and national scale models will usually lack the resolution and accuracy needed to identify these clusters.

### Current FMD vaccination strategy and epidemiology

#### Adoption of ≥6PD_50_ vaccines and a two-dose primary course

Since this study was performed the vaccination policy in Turkey has changed and all cattle are now routinely vaccinated every six-months using ≥6PD_50_ vaccines with a two-dose primary course. This strategy has been accompanied by a dramatic reduction in reported outbreaks (about 1000 in 2013, 253 in 2014, and 263 in 2015^28^,^29^,^30^,^31^). However, in late 2015 there was a rapid and widespread epidemic^32^ following the introduction of a new serotype A strain, prompting the production of a better matched vaccine^32^,^33^.

#### Impact of the new control policy

Although a decline in incidence is consistent with programme impact, epidemic cycles are typical in the region^34^ and a simple assessment of national incidence cannot separate the impact of natural and vaccine immunity^2^. Long-term impact is uncertain as although improved vaccination should improve disease control, FMD virus is highly infectious, pockets of high susceptibility are unavoidable and further incursions of new viruses, against which the vaccine may not protect, are likely due to cross-border animal movements.

### Interpretation and limitations

#### Natural immunity

Immunity derived from natural infection was not considered as it could confound the assessment of vaccine protection. For example, having low levels of vaccine immunity increases the likelihood of infection and thus natural immunity. Therefore, areas where the control programme is ineffective could be masked.

Overall population immunity, considering both vaccination and natural infection, can be assessed using representative sero-surveys^35^,^36^,^37^. However, this approach cannot distinguish whether immunity is derived from infection or vaccination, or both, and therefore, has limitations as a tool for evaluating a vaccination programme in endemic countries.

Including natural immunity in this modelling study would have been challenging. The epidemiology of FMD in Turkey is complex involving multiple viral strains and host species. Nationwide, group-specific estimates of virus exposure and immune response would be highly speculative in Anatolian Turkey, where many outbreaks are not reported and levels of under-reporting are uncertain and variable. In addition, estimates would have to consider the complexities of strain cross-protection, synergies between vaccine and viral antigen exposure and variable reactivity to the assays used to measure immunity. Most importantly for this study, this approach would not answer the question at hand, namely, what level of protection is provided by the vaccination strategy?

#### Maternal antibody

An additional simplification was the exclusion of maternally derived antibody. Looking at cattle with no prior infection, aged ≤7 months old, the maximum age when maternal protection was detected^13^, field studies of the evaluated vaccine^8^ found that from 23 unvaccinated calves 13%, 4% and 13% had an LPBE SP titre ≥1:10^2^ for serotypes A, O and Asia-1 respectively, with a further 13%, 9% and 17% that were borderline. The extent to which this maternal immunity is derived from vaccination as opposed to natural infection is uncertain and, hence, was not incorporated in this study. Furthermore, the relationship between antibody levels and protection is not known for young calves and may differ to that seen for older cattle^13^. Nonetheless, vaccine derived protection in young cattle ≤7 months old will be greater than reported here.

#### Categorisation of age and number of vaccine doses

The model simplification, whereby adult cattle were evenly distributed between ages 2–5 years old, would have minimal impact on estimates of the proportion vaccinated or protected, as adult cattle with multiple vaccinations (≥3 doses) were treated as one group and few cattle are >5 years old^13^. As seen in other studies^3^, increased immunity from additional vaccination beyond three doses was not evident in the field data^8^, as long as cattle were recently vaccinated. However, data on the immunity of old cattle, vaccinated many times were limited^8^ and serological predictions for cattle <24 months of age were more robust.

#### Death rates

Due to limitations in available data, death rates were not explicitly incorporated into the population model. However, variation in the rates at which older cattle are removed could affect seasonal variation in population immunity. To minimise this inaccuracy, as the census data used were collected in December, evaluations were also performed around this same period (Sept to March).

#### Relating antibody levels to protection

Although the exact interpretation of a serological protection threshold is uncertain, it provides a useful benchmark measure of immunity. We assessed serological thresholds at which 70% of cattle are protected against generalised FMD lesions. However, these thresholds are derived after vaccine-homologous virus is injected into the tongue and protection against field challenge may be greater^16^, although this would also depend upon vaccine match^38^,^39^.

The low VN titres seen for Asia-1 vaccine-homologous virus were surprising and may reflect within-serotype strain differences in the relationship between serology and protection reported by some^17^,^18^,^40^ but not all studies^15^. Ultimately, predictions will be influenced by the antigenic match between the vaccine, the test and the challenge virus.

#### Strategies assessed

Assessment of more scenarios would have been useful, including the use of higher potency vaccines or yearly vaccination of adult cattle. Unfortunately, the required immune response data were not available. The ≥6PD_50_ vaccine subsequently used for mass vaccination in Turkey was not yet available and although two studies had reported the long-term antibody response after a single dose of ≥6PD_50_ vaccine^7^,^27^, using these data was thought too speculative. Different vaccines, even with the same potency, have been seen to provide different levels of immunity and protection^41^. Vaccines used for FMD eradication in Europe stimulated high and long-lasting antibody levels for years after cattle had been vaccinated several times^3^,^42^,^43^, however, this may not be the case for other vaccines.

Limitations and assumptions are further discussed in the Supplementary Discussion.

### Findings in context

The findings of limited and variable vaccine protection are supported by other studies. FMD incidence is high in Anatolia with significant temporal, age and regional variation; surveys typically find about 15–20% of 6–18 month old cattle have serological evidence of prior infection^30^,^44^. Outbreak investigations have found variable vaccine coverage and although vaccination reduced the risk of FMD, incidence in vaccinated cattle was still high (35%)^13^.

In a process that took many decades, FMD has been controlled successfully using vaccination in South America^45^ and Europe^46^. However, when comparing this to FMD control in Anatolia there are many additional factors, besides vaccine protection, that must be considered. Levels of livestock mixing are high in Anatolia as most farms are smallholdings, densely concentrated within villages and dependent upon local and distant communal grazing. Levels of livestock movements are high, particularly around the Kurban festival of slaughter which involves the transportation of a fifth of all cattle and sheep (personal communication A.N. Bulut). Application of biosecurity measures is limited in Anatolia and as most smallholders require access to communal grazing, enforcing movement restrictions during outbreaks is challenging. Furthermore, extensive farmers in endemic regions may be less concerned about FMD outbreaks and less motivated to participate in control programmes^47^, this is compounded by the logistical challenges encountered when vaccinating many transhumant smallholders. Finally, livestock carrying new FMD viruses can enter Turkey from neighbouring endemic countries.

The importance of using vaccines with independent quality assurance and proven potency cannot be emphasised enough. If vaccination is ineffective, veterinary services and farmers bear the burden of mass vaccination without reducing the burden of disease. Furthermore, loss of confidence in vaccination and reduced participation in future control programmes can have long lasting repercussions.

## Conclusion

Low population immunity after FMD mass vaccination results from a) rapid antibody decay in vaccinated animals and b) a high proportion of animals that have not received a sufficient number of vaccine doses. Using higher potency vaccines and a two-dose primary course would result in longer lasting antibody titres being obtained at a younger age.

As most cattle have only been vaccinated ≤2 times, a more potent vaccine able to deliver greater immunity after only one or two doses would greatly increase population immunity. Immunity gaps will still exist as each round of mass vaccination is likely to exclude a quarter of all cattle. Prioritising repeated vaccination of young cattle, with high coverage, would help reduce this gap.

However, over-reliance on vaccination with limited movement controls or isolation of infected animals is not recommended as FMD virus is highly infectious and vaccine protection will still leave clusters of high susceptibility^7^,^13^,^48^. Susceptibility is exacerbated if there is a high risk of exposure to new virus strains, against which the vaccine may not protect.

In many FMD-endemic countries livestock movement restrictions and biosecurity measures are difficult to implement. In this situation FMD control becomes heavily dependent upon vaccine protection. However, the extent to which FMD can be controlled by vaccination alone remains an unanswered question of global importance.

## Additional Information

**How to cite this article**: Knight-Jones, T. J. D. *et al.* Mass vaccination, immunity and coverage: modelling population protection against foot-and-mouth disease in Turkish cattle. *Sci. Rep.* **6**, 22121; doi: 10.1038/srep22121 (2016).

## Supplementary Material

Supplementary Information

Supplementary Dataset

## Figures and Tables

**Table 1 t1:** Median predicted proportion of cattle vaccinated during mass FMD vaccination in Turkey [with 95% PI in brackets], allowing for incomplete coverage of eligible cattle assessed six months after the last round of vaccination**.

Number of doses			0	1	2	≥3	Total
Evaluated 25^th^ Sept 2012†	Months since last vaccination	Unvaccinated	22% [21.9–22.2]	0%	0%	0%	22% [21.9–22.2%]
		6	0%	10.4% [10.4–10.5]	13.1% [13.1–13.15]	39.4% [39.2–39.6]	62.9% [62.7–63.1]
		12	0%	3.19% [3.18–3.2]	1.87% [1.8–1.9]	6.7% [6.6–6.8]	11.8% [11.6–11.9]
		≥18	0%	0.77% [0.75–0.79]	0.7% [0.7–0.7]	1.85% [1.7–1.9]	3.3% [3.1–3.4]
	Age [months]	<6	13.1% [12.9–13.2]	0%	0%	0%	13.1% [12.9–13.2]
		6 -<12	6.8% [6.7–6.9]	4.4% [4.3–4.5]	0%	0%	11.2% [11.1–11.4]
		12 -<18	1.5% [1.4–1.6]	6.1% [6–6.3]	6.1% [6–6.2]	0%	13.7% [13.6–13.9]
		18 -<24	0.5% [0.5–0.6]	2.8% [2.7–2.9]	6% [5.9–6.1]	3% [2.9–3.1]	12.3% [12.2–12.5]
		≥24	0.1% [0.1–0.2]	1% [0.9–1.1]	3.5% [3.4–3.6]	44.9% [44.8–45]	49.6%*
		Total	22% [21.9–22.2]	14.5% [14.4–14.6]	15.6% [15.5–15.7]	47.9% [47.8–48.1]	100%
	If all eligible cattle always vaccinated		18.5% [18.4–18.6]	81.5% [81.4–81.6]	72.1% [72–72.2]	55.9% [55.7–56]	*Eligible that day 96.6% [96.5*–*96.65]*
Evaluated 25^th^ March 2013‡	Months since last vaccination	Unvaccinated	20% [19.8–20.2]	0%	0%	0%	20% [19.8–20.2]
		6	0%	14.4% [14.2–14.6]	10.3% [10.2–10.5]	41.3% [41.1–41.5]	66% [65.8–66.3]
		12	0%	2.4% [2.3–2.4]	2.4% [2.3–2.4]	6.7% [6.5–6.7]	11.4% [11.2–11.6]
		≥18	0%	0.7% [0.7–0.8]	0.4% [0.4–0.45]	1.4% [1.4–1.5]	2.6% [2.5–2.7]
	Age [months]	<6	11.2% [11–11.3]	0%	0%	0%	11.2% [11–11.3]
		6 -<12	5.6% [5.5–5.7]	7.5% [7.4–7.7	0%	0%	13.2% [13–13.3]
		12 -<18	1.8% [1.7–1.8]	6.6% [6.5–6.7]	3.8% [3.7–3.9]	0%	12.1% [12–12.3]
		18 -<24	0.4% [0.4–0.5]	2.5% [2.5–2.6]	5.9% [5.8–6]	5% [4.9–5.1]	13.9% [13.8–14]
		≥24	0.1% [0.1–0.2]	1.1% [1–1.1]	3.6% [3.5–3.7]	44.8% [44.7–44.9]	49.6*
		Total	20% [19.8–20.2]	17.7% [17.5–17.9]	13.3% [13.2–13.5]	49.8% [49.7–50]	100%
	If all eligible cattle always vaccinated		14.5% [14.4–14.6]	85.5% [85.4–85.6]	69.9% [69.8–70]	60% [59.8–60.1]	*Eligible that day 94.7% [94.6*–*94.8]*

Autumn revaccination (25th September 2012) would vaccinate 74.5% [95% PI: 74.3–74.8%] of eligible cattle, spring revaccination (25th March 2013) would vaccinate 73.2% [95% PI: 73–73.4%] of eligible cattle. The median total does not necessarily equal the total of the subcategory medians. This, along with rounding, resulted in minor discrepancies with margin totals.

^*^Fixed sampling proportion used from census data.

^**^District level coverage was described by a Betapert distribution (minimum = 40%, maximum = 100%, most likely = 80%).

^†^i.e. Assessed on day of autumn 2012 vaccination BEFORE vaccination performed.

^‡^i.e. Assessed on day of spring 2013 vaccination BEFORE vaccination performed.

**Table 2:** Regression coefficients from the two-step regression modelling process used to predict post-vaccination SP titre of cattle fitted to data from a prospective field study in Turkey (October 2012–March 2013)^8^.

Regression model	Parameter	Serotype O		Serotype A		Serotype Asia-1	
		Coefficient [95% CI]	P	Coefficient [95% CI]	P	Coefficient [95% CI]	P
Step 1: (Logistic) Titre ≥1:32 n = 1045	Intercept [vaccinated with single dose]	2.1 [1.8–2.5]	<0.001	0.6 [0.3–0.8]	<0.001	2.3 [1.9–2.7]	<0.001
	Prior vaccination [effect per dose 0-≥2]†	0.5 [0.09–0.9]	0.02	0.4 [0.1–0.7]	0.009	0.2 [−0.2–0.6]	0.4
	Time since last vaccination [effect per day]	−0.01 [−0.02 to −0.01]	<0.001	−0.006 [−0.009 to −0.003]	<0.001	−0.01 [−0.02 to −0.007]	<0.001
	Two-dose primary course given	1.4 [0.8–2]	<0.001	1.2 [0.9–1.6]	<0.001	1.3 [0.7–1.8]	<0.001
Step 2: (Linear) Log_10_(SP titre)	Intercept [vaccinated with single dose]	2.25 [2.2–2.3]	<0.001	2.1 [2–2.2]	<0.001	2.3 [2.25–2.4]	<0.001
	Prior vaccination [effect per dose 0-≥2]†	0.06 [0.01–0.1]	0.02	0.05 [−0.001–0.1]	0.08	0.06 [0.02–0.1]	0.007
	Time since last vaccination [effect per day]*	−0.001 [−0.002 to −0.0006]	<0.001	−0.0005 [−0.001 to 0.0003]	0.2	−0.002 [−0.003 to −0.001]	<0.001
	Two-dose primary course given	0.2 [0.14–0.26]	<0.001	0.11 [0.06–0.17]	<0.001	0.17 [0.1–0.2]	<0.001
	n [excludes cattle with titre <1:32]	843		638		887	

All unvaccinated cattle were given a zero titre. In step one, vaccinated cattle were assessed for their likelihood of having a detectable titre (≥1:32) using a GEE model with a logistic link function (log Odds Ratios are reported). In step two, the Log_10_(SP titre) was modelled in cattle with titres ≥1:32 using interval linear regression to allow for censoring in the data. 95% confidence intervals [95% CI] are reported calculated from robust standard errors, allowing for repeat sampling of individual cattle.

Unvaccinated cattle were given a titre of zero.

^*^An exponential decay function, as Log_10_(SP titre) is modelled.

^†^Age was excluded as it was collinear with number of prior vaccine doses.

**Table 3:** Median predicted proportion of the Turkish cattle population with an FMD Log_10_(Sp titre) of <2 and zero (below 1:32 dilution detection threshold), stratified by age [95% PI in brackets].

Serotype	Log_10_ SP titre	Age [months]	October	February	
			One-dose primary	One-dose primary	Two-dose primary
O	<2	<6	72% [69–73]	100% [100–100]	100% [100–100]
		6 -<18	45% [42–48]	76% [71–80]	51% [47–56]
		18 -<24	38% [33–44]	63% [55–71]	57% [48–67]
		≥24	34% [28–42]	59% [48–69]	58% [48–69]
		Total	42% [38–47]	68% [60–75]	60% [53–68]
	0	<6	62% [60–63]	100% [100–100]	100% [100–100]
		6 -<18	28% [26–31]	58% [52–65]	38% [33–43]
		18 -<24	22% [18–27]	44% [35–54]	38% [27–51]
		≥24	19% [13–26]	39% [27–52]	38% [26–52]
		Total	27% [23–31]	51% [43–59]	44% [35–54]
A	<2	<6	84% [83–86]	100% [100–100]	100% [100–100]
		6 -<18	64% [60–68]	81% [76–85]	63% [58–68]
		18 -<24	55% [48–63]	70% [60–78]	64% [53–74]
		≥24	51% [41–61]	65% [52–76]	64% [52–75]
		Total	59% [52–67]	73% [65–80]	68% [58–68]
	0	<6	73% [71–76]	100% [100–100]	100% [100–100]
		6 -<18	45% [41–49]	66% [60–71]	46% [41–51]
		18 -<24	35% [28–44]	51% [40–62]	44% [32–58]
		≥24	30% [22–42]	45% [31–59]	44% [30–59]
		Total	40% [34–47]	56% [47–66]	50% [40–61]
Asia-1	<2	<6	69% [67–71]	100% [100–100]	100% [100–100]
		6 -<18	42% [39–46]	74% [69–79]	53% [49–58]
		18 -<24	37% [32–44]	66% [58–74]	62% [52–71]
		≥24	35% [29–43]	63% [52–73]	63% [52–73]
		Total	41% [37–47]	70% [62–76]	64% [56–71]
	0	<6	60% [59–62]	100% [100–100]	100% [100–100]
		6 -<18	26% [24–29]	49% [43–56]	36% [31–41]
		18 -<24	22% [17–29]	41% [30–52]	38% [25–52]
		≥24	20% [14–20]	39% [25–54]	39% [25–54]
		Total	27% [23–33]	48% [38–58]	44% [33–54]

Estimates are made for one month after autumn vaccination (October) and one month before revaccination (February). Antibody levels in February were assessed with and without the routine use of a two-dose primary vaccination course (labelled “Two-dose”, and “One-dose” respectively). See table S1 for age distributions.

## References

[b1] Knight-Jones T. J. D. & Rushton J. The economic impacts of foot and mouth disease-what are they, how big are they and where do they occur? Prev Vet Med 112, 161–173, 10.1016/j.prevetmed.2013.07.013 (2013). 23958457 PMC3989032

[b2] Knight-Jones T. J. D., Edmond K., Gubbins S. & Paton D. J. Veterinary and human vaccine evaluation methods. Proc Biol Sci 281, 20132839, 10.1098/rspb.2013.2839 (2014). 24741009 PMC4043076

[b3] Terpstra C., Van Maanen C. & Van Bekkum J. G. Endurance of immunity against foot-and-mouth disease in cattle after three consecutive annual vaccinations. Res Vet Sci 49, 236–242 (1990). 2173088

[b4] Cox S. J. *et al.* Longevity of protection in cattle following immunisation with emergency FMD A22 serotype vaccine from the UK strategic reserve. Vaccine 28, 2318–2322, 10.1016/j.vaccine.2009.12.065 (2010). 20056183

[b5] Doel T. R. FMD vaccines. Virus Res 91, 81–99 (2003). 12527439 10.1016/s0168-1702(02)00261-7

[b6] Brehm K. E., Kumar N., Thulke H. H. & Haas B. High potency vaccines induce protection against heterologous challenge with foot-and-mouth disease virus. Vaccine 26, 1681–1687, 10.1016/j.vaccine.2008.01.038 (2008). 18313814

[b7] Pay T. W. F. Factors influencing the performance of foot-and-mouth disease vaccines under field conditions In. Applied virology, (ed Kurstak E.) 73–86 (Academic Press Inc. Orlando, USA & London, 1984).

[b8] Knight-Jones T. J. D. *et al.* Randomised field trial to evaluate serological response after foot-and-mouth disease vaccination in Turkey. Vaccine 33, 805–811, 10.1016/j.vaccine.2014.12.010 (2015). 25528523 PMC4334422

[b9] Doel T. R. Natural and vaccine induced immunity to FMD. Curr Top Microbiol 288, 103–131 (2005). 10.1007/3-540-27109-0_5 15648176

[b10] Späth E. J. *et al.* Immune response of calves to foot-and-mouth disease virus vaccine emulsified with oil adjuvant. Strategies of vaccination. Vaccine 13, 909–914, 10.1016/0264-410X(95)00009-P (1995). 7483763

[b11] Turkish Statistical Institute. Animal Production Statistics, 2012- http://www.turkstat.gov.tr/PreHaberBultenleri.do? id=13512. (2013) (Date of access: 01/11/2013).

[b12] Askaroglu H. EU project for the control of FMD in Turkey, West Eurasia Roadmap FMD Control 2010-2020, 7–9 October, 2009 – Istanbul, Turkey. (2009).

[b13] Knight-Jones T. J. D. *et al.* Retrospective evaluation of foot-and-mouth disease vaccine effectiveness in Turkey. Vaccine 32, 1848–1855, 10.1016/j.vaccine.2014.01.071 (2014). 24530150 PMC3991324

[b14] Reeve R. *et al.* Reducing animal experimentation in foot-and-mouth disease vaccine potency tests. Vaccine 29, 5467–5473, 10.1016/j.vaccine.2011.05.056 (2011). 21640777

[b15] Barnett P. V., Statham R. J., Vosloo W. & Haydon D. T. Foot-and-mouth disease vaccine potency testing: determination and statistical validation of a model using a serological approach. Vaccine 21, 3240–3248, 10.1016/s0264-410x(03)00219-6 (2003). 12804854

[b16] Van Bekkum J. G., Fish R. C. & Nathans I. Immunologic responses in dutch cattle vaccinated with foot-and-mouth disease vaccines under field conditions: Neutrailizing antibody responses and immunity to O, A and C types. Am J Vet Res 30, 2125–2129 (1969). 5389419

[b17] Pay T. W. F. & Hingley P. J. The use of serum neutralizing antibody assay for the determination of the potency of foot and mouth disease (FMD) vaccines in cattle. Dev Biol Stand 64, 153–161 (1986). 3025036

[b18] Van Maanen C. & Terpstra C. Comparison of a liquid-phase blocking sandwich ELISA and a serum neutralization test to evaluate immunity in potency tests of foot-and-mouth disease vaccines. J Virol Methods 124, 111–119 (1989). 10.1016/0022-1759(89)90192-0 2553819

[b19] Gelman A. & Hill J. Data analysis using regression and multilevel/hierarchical models. Pp 150–1, (Cambridge University Press, 2007).

[b20] Smitsaart E. N. *et al.* Assessment using ELISA of the herd immunity levels induced in cattle by foot-and-mouth disease oil vaccines. Prev Vet Med 33, 283–296 (1998). 9500182 10.1016/s0167-5877(97)00014-7

[b21] Robiolo B. *et al.* Confidence in indirect assessment of foot-and-mouth disease vaccine potency and vaccine matching carried out by liquid phase ELISA and virus neutralization tests. Vaccine 28, 6235–6241, 10.1016/j.vaccine.2010.07.012 (2010). 20643090

[b22] Robiolo B. *et al.* Analysis of the immune response to FMDV structural and non-structural proteins in cattle in Argentina by the combined use of liquid phase and 3ABC-ELISA tests. Vaccine 24, 997–1008, 10.1016/j.vaccine.2005.08.071 (2006). 16171905

[b23] R Core Team. R: A language and environment for statistical computing. R Foundation for Statistical Computing, Vienna, Austria. URL http://www.R-project.org/. (2014) (Date or access: 25/11/2015).

[b24] Barasa M. *et al.* Foot-and-Mouth Disease Vaccination in South Sudan: Benefit–Cost Analysis and Livelihoods Impact. Transbound Emerg Dis 55, 339–351, 10.1111/j.1865-1682.2008.01042.x (2008). 18786073

[b25] Forman S. *et al.* Moving towards the global control of foot and mouth disease: an opportunity for donors. Rev Sci Tech 28, 883–896 (2009). 20462147 10.20506/rst.28.3.1935

[b26] Sutmoller P., Barteling S. S., Olascoaga R. C. & Sumption K. J. Control and eradication of foot-and-mouth disease. Virus Res 91, 101–144 (2003). 12527440 10.1016/s0168-1702(02)00262-9

[b27] Selman P., Chénard G. & Dekker A. Cedivac-FMD; Duration of Immunity in cattle, sheep and pigs. Open session of the EuFMD, Paphos, Cyprus, 17–19 October 2006 http://www.fao.org/ag/againfo/commissions/docs/research_group/paphos/App31.pdf. (2006) (Date of access: 21/02/2014).

[b28] Bulut A. N. FMD situation in Turkey presented at 6th West Eurasia Roadmap Meeting, Almaty, Kazakhstan, 28-30 April 2015 http://www.fao.org/fileadmin/user_upload/eufmd/Roadmap_2015/TurkeyCountryReport.pdf. (2015) (Date of access: 25/11/2015).

[b29] Bulut A. N. FMD situation in Turkey-5th West Eurasia roadmap meeting. Astana, Kazakhstan 23–24 April 2014. (2014).

[b30] Bulut A. N. FMD situation in Turkey. West EurAsia FMD Control-Roadmap 2025, 4th Annual Regional Progress Review Meeting, 2–4th April 2013, Baku, Azerbaijan. (2013).

[b31] EC-ADNS. Animal disease notification system: outbreaks per disease ADNS disease overview report 1st Jan–31st Dec 2015 http://ec.europa.eu/food/animal/diseases/adns/table_11_2015.pdf. (2015) (Date of access: 07/01/2016).

[b32] ProMED. Foot and mouth disease-Turkey (06): serotype A, topotype asia, Genotype VII, spread, endemic, oie, request for information. Report Wed 16 Dec 2015 Archive Number: 20151218.3872002 http://www.promedmail.org/post/3872002. (2015) (Date of access: 07/01/2016).

[b33] The Pirbright Institute & The Sap Institute. WRLFMD-Joint Genotyping Report with the Sap Institute. Turkey FMDV type A 10 November 2015 http://www.wrlfmd.org/fmd_genotyping/2015/WRLMEG-2015-00019%20A%20Turkey%202015%20v2.pdf. (2015) (Date of access: 07/01/2016).

[b34] Knight-Jones, T. J. D. PhD Thesis-Field evaluation of foot-and-mouth disease vaccination in Turkey. The Pirbright Institute & The Royal Veterinary College, University of London. https://www.researchgate.net/publication/271498205_Field_evaluation_of_foot-and-mouth_disease_vaccination_in_Turkey, (2014) (Date of access: 25/11/2015).

[b35] Metwally S. *et al.* OIE-FAO Foot and mouth disease post-vaccination monitoring guidelines. (In press).

[b36] Robiolo B. *et al.* Quantitative single serum-dilution liquid phase competitive blocking ELISA for the assessment of herd immunity and expected protection against foot-and-mouth disease virus in vaccinated cattle. J Virol Methods 166, 21–27, 10.1016/j.jviromet.2010.02.011 (2010). 20170683

[b37] León E. A. *et al.* Effectiveness of systematic foot and mouth disease mass vaccination campaigns in Argentina Rev Sci Tech 33, 917–926 (2014). 25812215 10.20506/rst.33.3.2329

[b38] Arrowsmith A. E. Variation among strains of type A foot-and-mouth disease virus in the Eastern Mediterranean region 1964–1972. J Hyg (Lond) 75, 387–397 (1975). 172558 10.1017/s0022172400024451 PMC2130354

[b39] Knowles N. J. *et al.* Recent spread of a new strain (A-Iran-05) of foot-and-mouth disease virus type A in the Middle East. Transbound Emerg Dis 56, 157–169, 10.1111/j.1865-1682.2009.01074.x (2009). 19432637

[b40] Maradei E. *et al.* Updating of the correlation between lpELISA titers and protection from virus challenge for the assessment of the potency of polyvalent aphtovirus vaccines in Argentina. Vaccine 26, 6577–6586, 10.1016/j.vaccine.2008.09.033 (2008). 18835312

[b41] Robinson L. & Knight-Jones T. J. D. Global FMD Research Alliance/EuFMD-Global foot-and-mouth disease research update and gap analysis 2014-Vaccines chapter http://go.usa.gov/cZgG9. (2014) (Date of access: 25/01/2016).

[b42] Dekker A. & Terpstra C. Prevalence of foot-and-mouth disease antibodies in dairy herds in The Netherlands four years after vaccination. Res Vet Sci 61, 89–91 (1996). 8819202 10.1016/s0034-5288(96)90118-6

[b43] Remond M., Kaiser C., Lebreton F. O., Moutou F. & Cruciere C. Residual foot-and-mouth disease virus antibodies in French cattle and sheep six years after the vaccination ban. Vet Res 32, 81–86, 10.1051/vetres:2001112 (2001). 11254180

[b44] McLaws M. Monitoring FMD occurance-EuFMD Open session, 29–31 Oct 2012, Jerez, Spain (2012).

[b45] Naranjo J. & Cosivi O. Elimination of foot-and-mouth disease in South America: lessons and challenges. Philos Trans R Soc Lond B Biol Sci 368, 20120381, 1098/rstb.2012.0381 (2013). 23798699 10.1098/rstb.2012.0381 PMC3720049

[b46] Leforban Y. & Gerbier G. Review of the status of foot and mouth disease and approach to control/eradication in Europe and Central Asia. Rev Sci Tech 21, 477–492 (2002). 12523688 10.20506/rst.21.3.1345

[b47] Knight-Jones T. J. D. & Rushton J. Foot-and-mouth disease impact on smallholders-What do we know, what don’t we know and how can we find out more? Transbound Emerg Dis In press (2016). 10.1111/tbed.12507 PMC5516236 27167976

[b48] Woolhouse M. E. J., Haydon D. T., Pearson A. & Kitching R. P. P. Failure of vaccination to prevent outbreaks of foot and mouth disease. Epidemiol Infect 116, 363–371 (1996). 8666082 10.1017/s0950268800052699 PMC2271420

