# Supplementary material for: Mass vaccination, immunity and coverage: modelling population protection against foot-and-mouth disease in Turkish cattle
Source: Sci Rep. 2016 Feb 26;6:22121. doi: 10.1038/srep22121 (PMC4768268; doi:10.1038/srep22121)

Mass vaccination, immunity and coverage: modelling population protection against foot-and-mouth disease in Turkish cattle

T. J. D. KNIGHT-JONES, S. GUBBINS, A. N. BULUT, K. D. C. STÄRK, D. U. PFEIFFER, K. J. SUMPTION, D. J. PATON

Supplementary information

Supplementary Methods

Coverage and protection

The relationship between percentage vaccinated and population immunity was explored graphically and with simple linear regression. For simplicity, this was only done using serotype O without the two-dose primary course, assessed in mid-February.

Supplementary Results

Coverage and protection

The relationship between the proportion vaccinated at the district level and the proportion with a protective titre was linear but there was substantial variation (Supplementary Fig. S4). This results from simultaneous variation in proportions vaccinated by number of doses, uncertainty, and random variation in immune response. Looking at immunity approximately five months after autumn vaccination, the model predicts that on average, for every one percent increase in cattle vaccinated ≥3 times in their lifetime an extra 0.4% of cattle in a district achieve an SP titre $\geq 1{:}10^2$ (R^2=31%). Irrespective of prior vaccination, each additional one percent vaccinated in a district at the last round of vaccination resulted in an additional 0.5% of cattle with a titre $\geq 1{:}10^2$ [Supplementary Fig. 4] (R^2=46%).

This suggests that for Turkey's 13.5 million cattle population, each one percent increase in the percentage vaccinated would increase population immunity at the national level by 0.5%. This

would cost about $0.135 million (at $1/dose), however, the cost of increasing coverage is unlikely to be linear.

Supplementary discussion

Model approaches, assumptions and limitations

Population structure was simulated assuming the number of cattle in each age category was the same as on 31st December 2012. Although, proportions within age-categories will change over the annual production cycle, this inaccuracy will be relatively small if assessment is done within a few months of the actual date that age-structure was determined as relatively few animals are slaughtered over winter [1]. Age-category specific estimates are unaffected.

Mass vaccination is typically conducted over many days. However, in order to simplify the model, vaccination was assumed to be conducted on a single day (the average date of vaccination was used). Including variation in vaccination date would result in a marginal amount of additional variation in estimates of post-vaccination population immunity without affecting average estimates.

Data describing variation in coverage by location or by production type were not available and district coverage was assumed to be randomly distributed. As a result, although the aggregated estimates for Turkey are valid, spatial variation in the proportion of eligible cattle that are vaccinated could not be incorporated. However, assessment assuming 100% of eligible cattle were vaccinated revealed how regional variation in demographics affected the proportion of the population vaccinated, and how this changes during the six month inter-vaccination period. Correlations between seasonal calving patterns and the proportion of eligible cattle vaccinated could not be modelled.

Interval regression was used to adjust for the interval and right censoring present in the original serial dilution serology data. Thus the predicted titre was not restricted by the dilutions used during testing but could take any dilution ≥0. A GEE approach was used with an exchangeable correlation matrix in step one and a working independence variance structure in step two

assuming independence of individual cattle. GEE models provide population averaged parameter estimates. This differs to random effects models, which provide cluster specific parameter estimates which are less suitable for the population level predictions required in this modelling study.

The model is based entirely on data (randomised surveys and demographic census) allowing for uncertainty and variation at all steps. The SP prediction model accurately described immunity levels. The large number of simulations and large data sets allowed precise estimates to be made of aggregated population immunity. However, if small sub-groups with extreme values were singled out there was a high risk of selecting values that arose by chance. For this reason, district level results were not assessed and individual provincial estimates should be interpreted with caution.

References

1 Knight-Jones, T. J. D. *et al.* Randomised field trial to evaluate serological response after foot-and-mouth disease vaccination in Turkey. *Vaccine* **33**, 805-811, doi:10.1016/j.vaccine.2014.12.010 (2015).

Supplementary table

Table S1: Simulated age distribution of the Turkish cattle population in October 2012 and February 2013. Median values are shown with 95% prediction intervals (PI) in brackets.

Age [months]	Age distribution	
	October	February
<6	11.8% [11.7-12]	9.7% [9.6-9.8]
6 - <12	12.5% [12.3-12.6]	14.6% [14.5-14.7]
12 - <18	12.5% [12.3-12.6]	10.4% [10.3-10.6]
18 - <24	13.6% [13.5-13.8]	15.6% [15.5-15.8]
≥24	49.6*	49.6%*
Total	100%	100%

*Fixed sampling proportion used from census data.

Supplementary figures

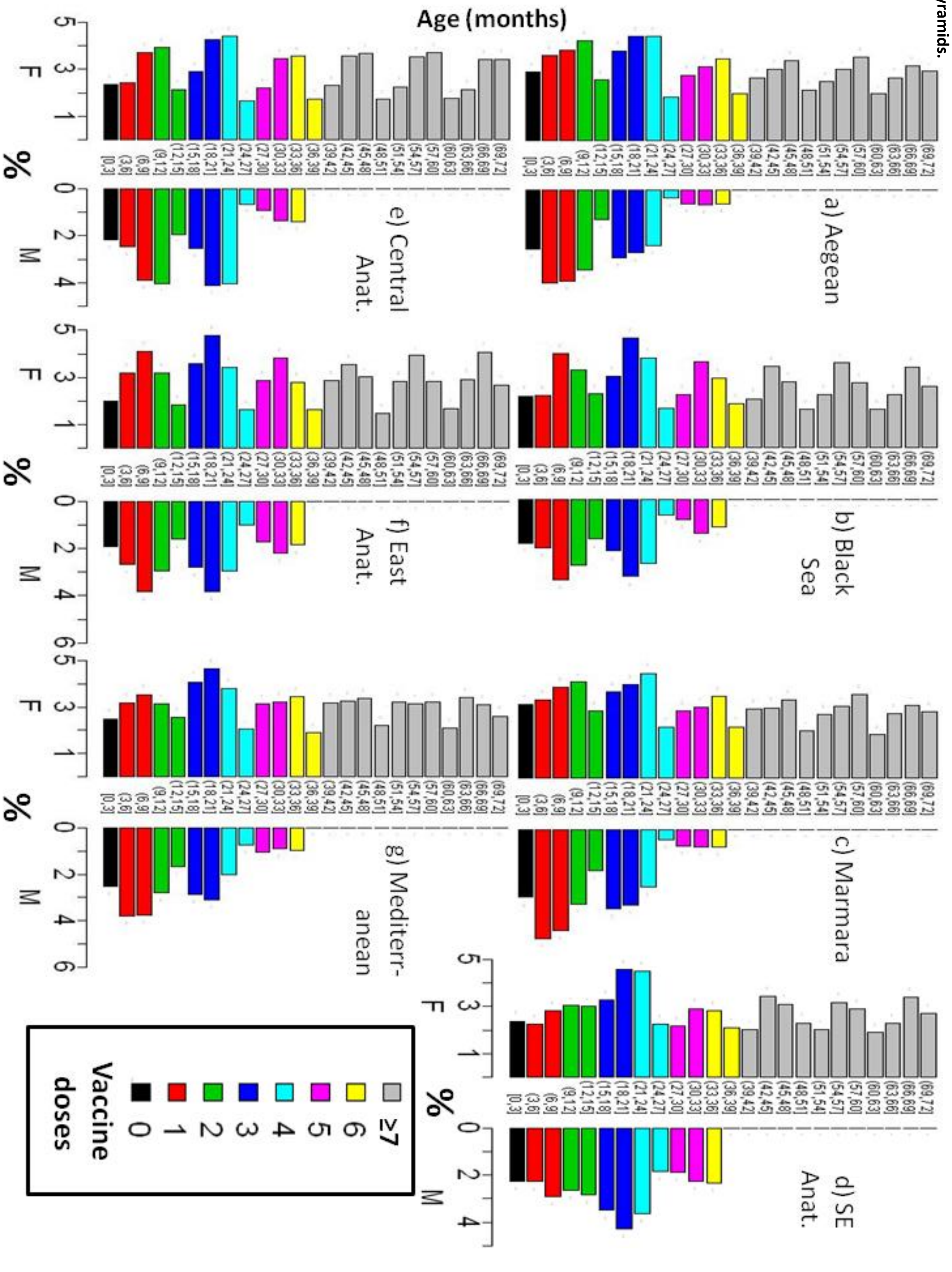

Figure S1: Population pyramids showing the mean, modelled cattle age by sex structure in the seven regions of Turkey on 31st Dec 2012. The number of vaccine doses cattle would have received in their lifetime if all eligible cattle were vaccinated in autumn and spring each year is colour coded. Exact year of birth of cattle >24 months has not been modelled precisely. [Anat.=Anatolia, M=Male, F=Female]. Aggregating age-groups has partially obscured seasonal birth patterns in the population pyramids.

Figure S2: Maps showing modelled percentage of Turkish cattle previously vaccinated, assessed at time of spring 2013 vaccination assuming all eligible cattle were vaccinated each spring and autumn. Maps show (above) the median percentage unvaccinated and (below) the median percentage vaccinated at least three times (the number of doses needed for a sustained antibody titre). Thrace is not included. Created using ArcGIS® software by Esri (ArcMAP10.3).

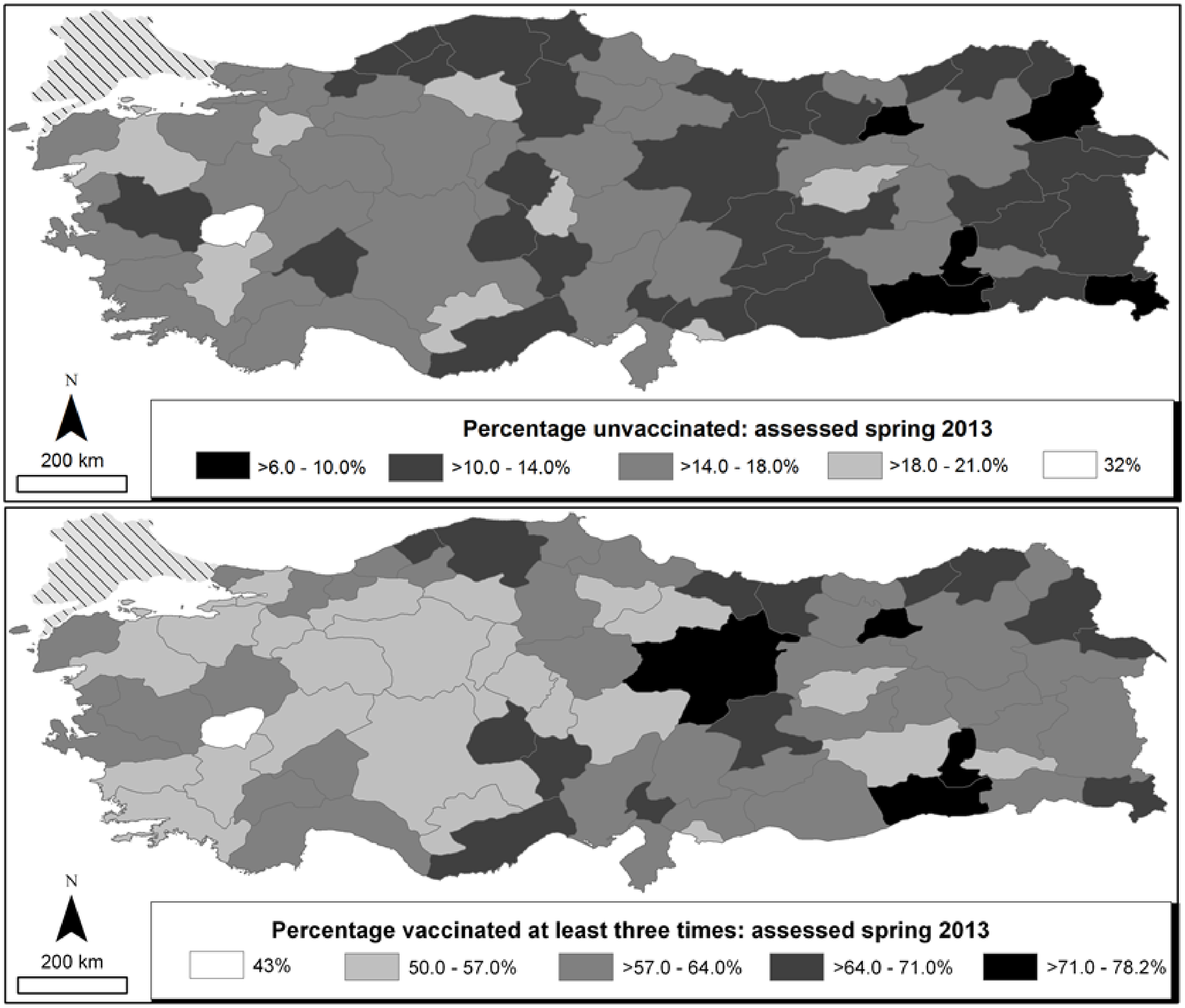

Figure S3: Predicted Log_{10}(LPBE SP titre) for the Turkish cattle population after September 2012 FMD vaccination. For serotypes O (top), A (middle) and Asia-1 (bottom). February 2013 titres (142 days post September vaccination) are shown with and without the routine use of a two-dose primary vaccination course (labelled "Two-dose", and "One-dose" respectively). The proportion of cattle with a particular titre is proportional to the width of the violin-plot. Within the violin-plot is a box-plot showing the median titre (white circle), inter-quartile range and minimum and maximum values. The "protection threshold" titre is indicated ($SP=1:10^2$). Although plots are shown for September and March, they are beyond the limits of the inter-vaccination interval in the data used to fit the prediction models [1] and are thus speculative.

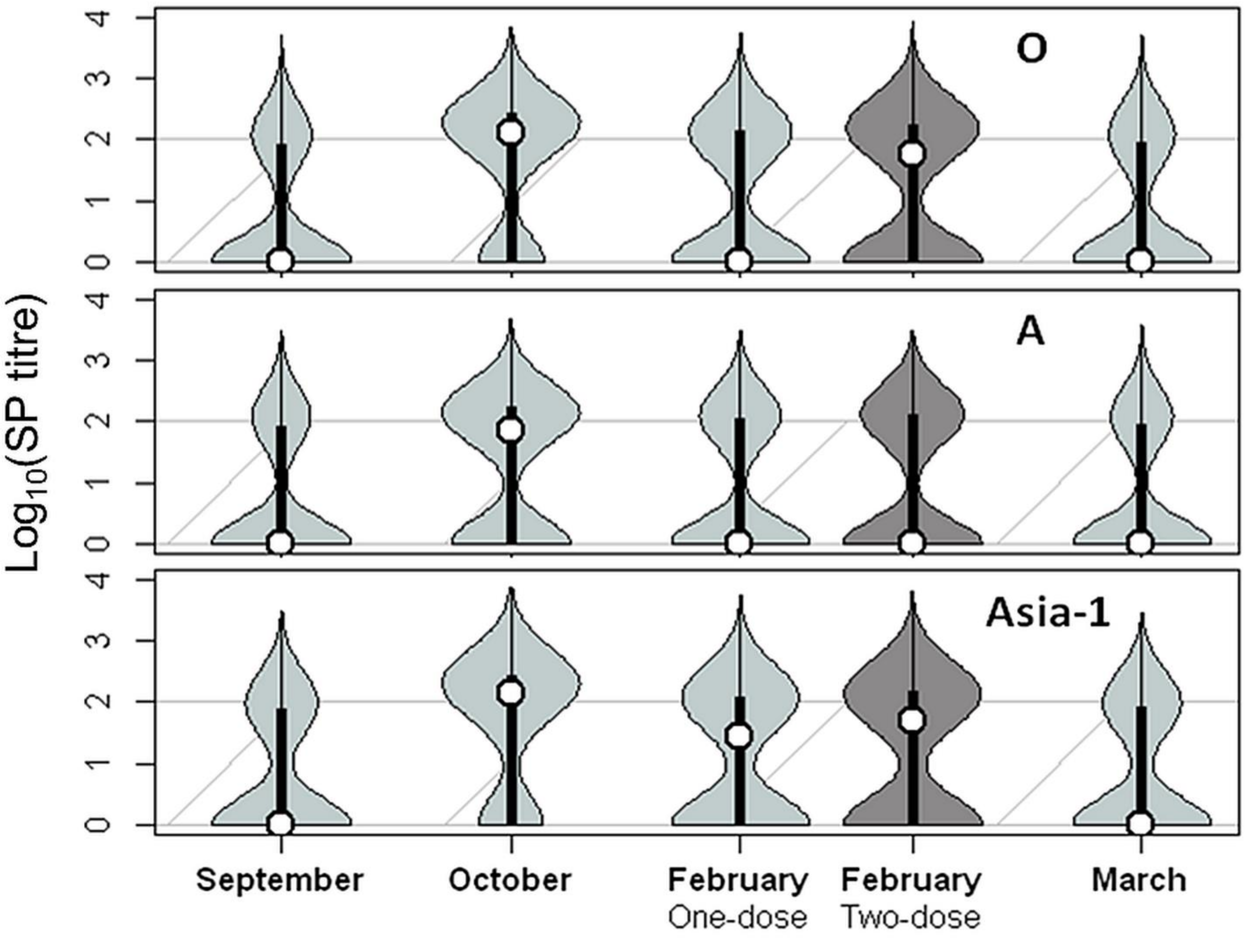

Figure S4: Modelled proportion vaccinated in a district at autumn 2012 mass vaccination against the percentage of cattle in a district with a serotype O SP titre ≥1:10^2 in mid-February 2013 (black solid line shows the median value, dashed lines show 5th and 95th percentiles, dotted lines show 2.5th and 97.5th percentiles).

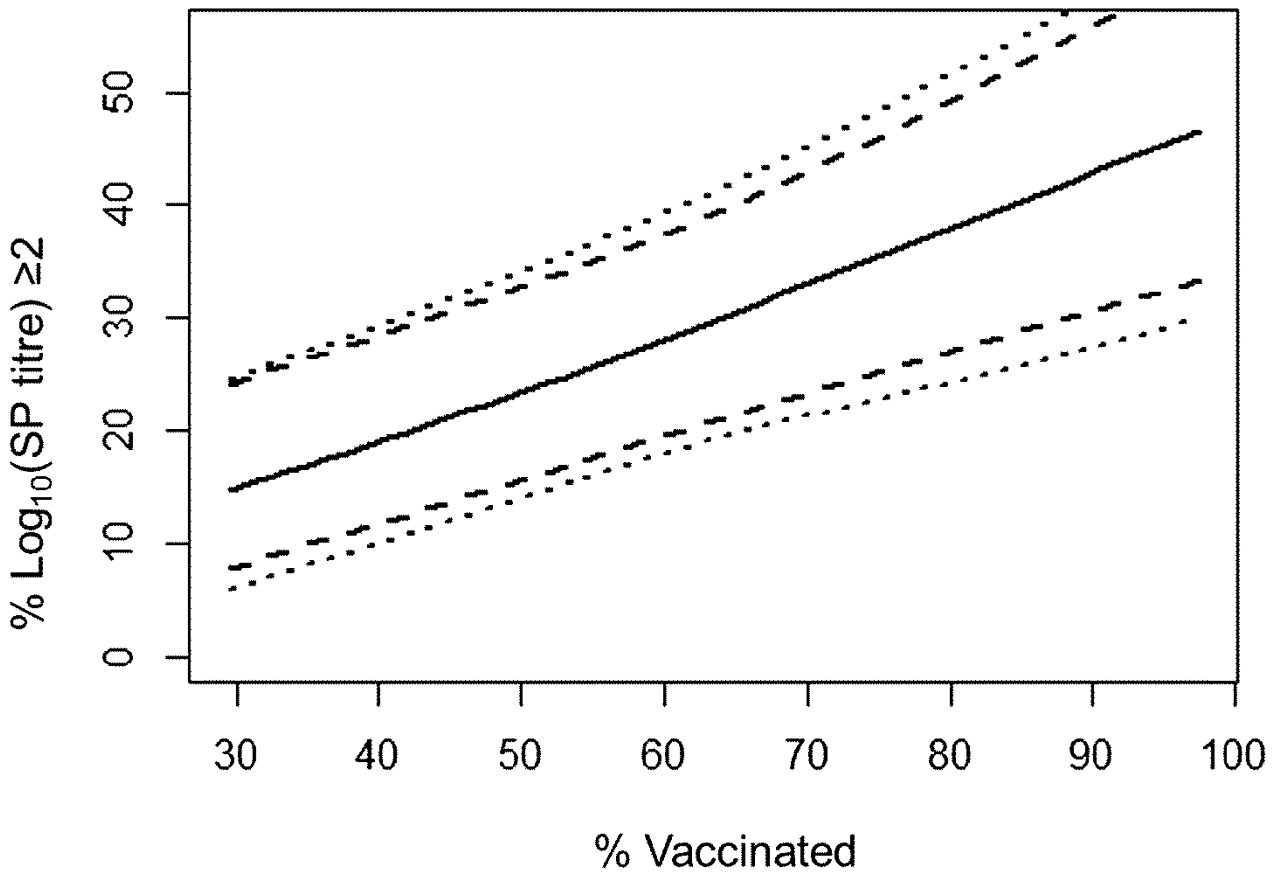

Supplement: Supplementary Information [file srep22121-s1.pdf]
